# The Projection of Iran’s Healthcare Expenditures By 2030: Evidence of a Time-Series Analysis

**DOI:** 10.34172/ijhpm.2022.5405

**Published:** 2022-02-01

**Authors:** Nader Jahanmehr, Mohammad Noferesti, Soheila Damiri, Zhaleh Abdi, Reza Goudarzi

**Affiliations:** ^1^Health Economics, Management and Policy Department, Virtual School of Medical Education & Management, Shahid Beheshti University of Medical Sciences, Tehran, Iran.; ^2^Department of Economics, School of Economics and Political Sciences, Shahid Beheshti University, Tehran, Iran.; ^3^Department of Health Management & Economics, School of Public Health, Tehran University of Medical Sciences, Tehran, Iran.; ^4^National Institute of Health Research, Tehran University of Medical Sciences, Tehran, Iran.; ^5^Health Services Management Research Center, Institute for Futures Studies in Health, Kerman University of Medical Sciences, Kerman, Iran.

**Keywords:** Health Expenditure, Health Insurance, Public Health Expenditure, Out-of-Pocket Payment, Health Financing, Iran

## Abstract

**Background:** The projection of levels and composition of financial resources for the healthcare expenditure (HCE) and relevant trends can provide a basis for future health financing reforms. This study aimed to project Iran’s HCEs by the sources of funds until 2030.

**Methods:** The structural macro-econometric modeling in the EViews 9 software was employed to simulate and project Iran’s HCE by the sources of funds (government health expenditure [GHCE], social security organization health expenditure [SOHCE], out-of-pocket [OOP] payments, and prepaid private health expenditure [PPHCE]). The behavioral equations were estimated by autoregressive distributed lag (ARDL) approach.

**Results:** If there is a 5%-increase in Iran’s oil revenues, the mean growth rate of gross domestic product (GDP) is about 2% until 2030. By this scenario, the total HCE (THCE), GHCE, SOHCE, OOP, and PPHCE increases about 30.5%, 25.9%, 34.4%, 31.2%, and 33.9%, respectively. Therefore, the THCE as a percentage of the GDP will increase from 9.6% in 2016 to 10.7% in 2030. It is predicted that Iran’s THCE will cover 22.2%, 23.3%, 40%, and 14.5% by the government, social security organization (SSO), households OOP, and other private sources, respectively, in 2030.

**Conclusion:** Until 2030, Iran’s health expenditures will grow faster than the GDP, government revenues, and non-health spending. Despite the increase in GHCE and total government expenditure, the share of the GHCE from THCE has a decreasing trend. OOP payments remain among the major sources of financing for Iran’s HCE.

## Background

**Key Messages**

** Implications for policy makers**

- Until 2030, the growth rate of health expenditures will be higher than the gross domestic product (GDP) and government revenues and expenditures.
- Government resources will not adapt to the rapid growth of health expenditures, and the government’s share in financing health expenditures will decrease.
- If the current trends continue, the Iranian health system will not successfully reduce the share of out-of-pocket (OOP) payments to less than 30% in the medium term (until 2030).
- Policy-makers should give special attention to efforts to slow the growth rate of health spending and efficient resource management.
- To avoid the impact of financial and economic crises on population health, financing policies should move towards options that ensure system resilience.

** Implications for the public**

All countries are working to ensure universal access to healthcare. Health financing system reform is the backbone of all efforts to achieve this goal. A correct understanding of the current level and composition of healthcare costs and their future direction is a useful guide to appropriate policies in this area. Over the next few years, health expenditure will grow faster than local incomes, threatening the stability of the healthcare financial system, government finances, and economic stability. In this situation, improving the efficiency of the health system and resource management should be pursued more seriously. In this situation, improving the efficiency of the health system and resource management can lead to increased compliance between revenue growth rates and expenditure growth rates and maintaining the stability of the health sector financial system.

The healthcare system is one of the determinants of health that participates with other factors in promoting population health. Without healthcare, there are many lost opportunities for significant improvements in population health.^1^ Therefore, guaranteeing the public access to necessary health services without any financial hardship – universal health coverage (UHC) – is put on top of the 2030 Agenda for Sustainable Development Goals.^2,3^ Achieve UHC requires robust healthcare systems with good governance and sustainable financing.^4^

Health financing concerns mobilization, accumulation, and allocation of money to cover healthcare needs.^5^ The fairly and effective mobilization of sufficient and sustainable financial resources for the health sector is the most significant purpose of health financing systems.^6^ However, the health policy-makers encounter to increase the challenges in achieving these goals around the world.^7^ The countries deal with the challenge of sufficient resources mobilization for health financing.^8^ Estimations indicate that out-of-pocket (OOP) payments are important in health financing, particularly among low- and middle-income countries. It is predicted to remain the main source of total healthcare expenditure (THCE) financing. These values are 12.7%, 29.6%, 51.2%, and 39.2% in high-income, upper-middle-income, lower-middle-income, and low-income countries, respectively, until 2050. In Iran, OOP payment was 37.6% in 2016 and 38.9% in 2050.^9^ Increased health expenditure along with an increased share of the THCE from gross domestic product (GDP) due to factors including technological advancement, demographic transition, increased expectations of consumers, and resources limitations resulted in the inability or unwillingness of the governments to increase spending on healthcare services which may threaten sustainable financing of health systems in the future.^10^

Improving the equity in health financing and financial protection was emphasized in Iran’s national developmental programs^11-16^ which was supported by numerous policies such as insurance program for patients on a hospital bed, free treatment of the victims of traffic accidents, development of the family physician program and rural insurance program, the establishment of boards of trustees in educational hospitals, and full-time geographical programs of physicians^17^. The Health Transformation Plan (HTP), which was launched in 2014, also sought to provide financial protection against health expenditures. HTP was funded through government resources, including a 59% increase in the annual budget of the Ministry of Health and Medical Education, the allocation of 10% of the resources obtained from the targeted subsidy plan, and 1% of value-added tax revenue to the health sector. However, Iran’s recent economic recession (difficulty selling oil due to imposed sanctions and the fluctuation of oil price) has prevented the full implementation of HTP. In other words, not receiving the approved budget by the public health insurance schemes imposed a massive debt on the health centers, so that the Ministry of Health and Medical Education was ordered to increase further the efficiency of health spending.^18,19^ Despite all these efforts, the evidence shows that financial protection has not yet been achieved among the Iranian population.^20,21^

The fiscal space for health reflects the government’s ability to increase health expenditure without any prejudice to the sustainability of its financial position. It depends on a suitable macroeconomic environment characterized by sustainable economic growth and low budget deficit.^22^ Nevertheless, the challenges such as severe economic fluctuations,^23^ the budget’s dependence on oil revenue,^24,25^ low tax efforts,^26^ imposed economic sanctions,^27^ an increasing budget deficiency,^28^ and other factors barriers to expanding fiscal space for health in Iran. For instance, the United States’ new sanctions have decreased Iran’s economic growth rate from 3.7% in 2017 to -4.8% in 2018.^29^ The failure of policies aims to improve financial protection, and the increasing growth of health expenditure and the above-mentioned economic problems can encounter Iran with many challenges to achieve the UHC in future. Up to the past three decades, the health expenditure has rapidly grown in many countries, including Iran, which are crucial concerns for policy-makers, patients, and insurance companies.^30-32^ From 1995 to 2016, the annual HCE increased by 4% worldwide from 3.5 to 8 trillion dollars.^33^ Iran’s healthcare expenditure (HCE) increased from 7 to 34 billion dollars during these 21 years, becoming 4.8 times larger.^33-47^ Policy-makers across the globe are concerned about the financial sustainability of public expenditure, increasing price of healthcare services, health sector, and financial pressures on patients and their families; therefore, they seek several ways to assess the extent of these problems and to develop political reform plan to reduce the pressure of growing costs.^48^ Such concerns have motivated the design of various models for predicting health expenditure in many countries.^33,35-47^ Predicting HCE and its funding sources are vital for effective policy-making in the health sector.^36^ The correct perception of the level and compositions of the resources for financing HCE and its trend over time can act as the basis for future health financing policy formulation.^49^ Considering this background, this study aimed to project Iran’s health expenditure until 2030.

## Methods

###  Data

A structural macro-econometrics model was employed to simulate and project Iran’s HCE. The THCE was divided into two components, ie, public health expenditure (including the government health expenditure [GHCE] and social security organization health expenditure [SOHCE]) and the private health expenditure (including OOP payments and prepaid private health expenditure [PPHCE]). For selecting the variables, we first reviewed the existing studies on the determinants of health expenditures and studies conducted to predict health expenditures. A set of variables was extracted and evaluated in several meetings of the research team members, and the variables that seemed appropriate to the study’s objectives entered the modeling process. Finally, in addition to the health expenditure variables, a wide range of variables, including economic, government revenue and expenditure, population variables, and health insurance variables employed to develop the structural macro model. The annual time series data of variables, extracted from the different databases such as Central Bank of the Islamic Republic of Iran, Statistical Center of Iran, statistical reports of the social security organization (SSO), statistical reports of the Central Insurance of Iran, and annual budget laws. additional information about data source of each variable are provided in Table S1 (see Supplementary file 1). However, some variables data were available from 1959. Since other data were unavailable, the final model was simulated on the data related to 1991-2016.

###  Econometrics Model

 The macro-structural econometric model used in this study, which is a kind of simultaneous equations system, was created in the following steps by EViews 9 software:

1. Determining the variables co-integration rank: If the time series variables used in estimating model coefficients are not stationary, the result may be a spurious regression. In econometrics, co-integration helps estimate a regression due to the levels of variables without any spurious regression. The concept of co-integration means two or more variables of a time series are linked to show a long-run equilibrium relationship. However, the time series itself might be unstable. They follow each other so well when their difference is stable. The augmented Dicky–Fuller test was used to analyze the stationarity of variables. The results of this test are presented in Table S2 (see Supplementary file 2).
2. Estimating the coefficients:The behavioral equations of the model were estimated due to autoregressive distributed lag (ARDL) method. ARDLs are standard least-squares regressions, which include the lags of both the dependent variable and explanatory variables as regressors. For instance, when the research goal is to estimate a long-term equilibrium relationship between Y, X, and Z, the corresponding ARDL technique is defined as follows:
$$Y_{t}=\alpha_{0}+\sum_{J=1}^{P}\alpha_{j}Y_{t-j}+\sum_{J=1}^{Q1}\beta_{1j}X_{t-j}+\sum_{J=1}^{Q2}\beta_{2j}Z_{t-j}+V_{t}$$
3. Conducing Banerjee, Dolado, and Master tests: In ARDL equations, if the sum of coefficients of lag variables of the dependent variable is smaller than 1 ( $\sum_{i=1}a_{i}<1$ ), the dynamic model is inclined towards the long-run equilibrium model. Therefore, the following hypotheses are tested for the co-integration test.
$$\begin{array}{l} H0:\sum_{i=1}^{\rho }a_{i}-1\geq 0\\ H1:\sum_{i=1}^{\rho }a_{i}-1<0\\ Equation\;2.\;Hypotheses\;of\;the\;Co-Integration\;Test \end{array}$$
  The following equation calculates the t-statistic required to conduct the above test:
$$\begin{array}{l} t=\frac{\sum_{i=1}^{P}{\hat{a}}_{i}-1}{\sum_{i=1}^{\rho }s_{{\hat{a}}_{i}}}\\ Equation\;3.\;The\;Co-Integration\;Test\;Statistic \end{array}$$
  These statistics should be negative to confirm the existence of a long-run relationship. Its absolute value should be greater than the absolute value of critical quantity proposed by Banerjee, Dolado, and Master (1992).
4. Estimating the error correction model of each equation: If a long-run relationship is confirmed in ARDL equations, then it is possible to observe estimations of short-term and long-term relationships and the error correction coefficient of each equation in each equation in EViews 9 software. This error correction coefficient shows the speed of movement towards equilibrium.
5. Assumptions of the classical linear regression model assessment: Four tests of Jarque–Bera, Breusch–Godfrey, White, and Ramsey tests were utilized to check the normality of error terms distribution, lack of serial correlation between error terms and other variables, homoscedasticity of error term and accuracy of functional form specification, respectively.
6. Testing the Interactive performance of long-term equilibrium and short-term dynamisity relationships:To develop a model, each of the estimated equations should have enough conceptual and statistical validity and interact appropriately with each other. In other words, in-sample simulation results of a model should adequately match the historical trend of variables included in the model. Therefore, the dynamic simulation was performed by putting each estimated equation next to other model equations. Then, the root mean square error (RMSE) and the Utile index are employed to analyze the proximity of trends in generated variables to their historical trend. When the equation performance was evaluated inappropriate simulation, its specification was revised, and the model was again estimated using the ARDL technique. This process was repeated until it was possible to obtain a series of valid equations presenting acceptable simulation results. Then, this model was used for prediction. Demographic variables, urbanization rate, and oil revenues were considered exogenous in the adjusted model. The predictions of demographic variables were extracted from the world population prospect 2017 made by United Nations.^50^ Since oil revenues are affected by different national and international variables such as global supply and demand for oil, its prediction is complicated and beyond this research scope. Hence, the variable was considered exogenous compared to the equations system to analyze its effect based on scenario making. Various scenarios could be considered for the growth rate of Iran’s oil and gas revenues, including the growth rate of these revenues in the years after the Iran-Iraq war, the growth rate in the last 10 to 15 years, and any other possible scenario. However, this study uses predictions made by US Energy Information Administration.^51,52^ The average growth rate of 4.39% was predicted for the global oil price from 2016 to 2030.

## Results

This study aims to project Iran’s HCE until 2030 through a macro-econometrics model. The parameters of the equations included in this model, the standard deviation of the parameters, and other characteristics of the equations specified are presented in Supplementary file 3. Final developed dynamic macro-structural econometric model, the process of evaluating the validity of this model and related information is provided in Supplementary file 4.

**Figure 1:**
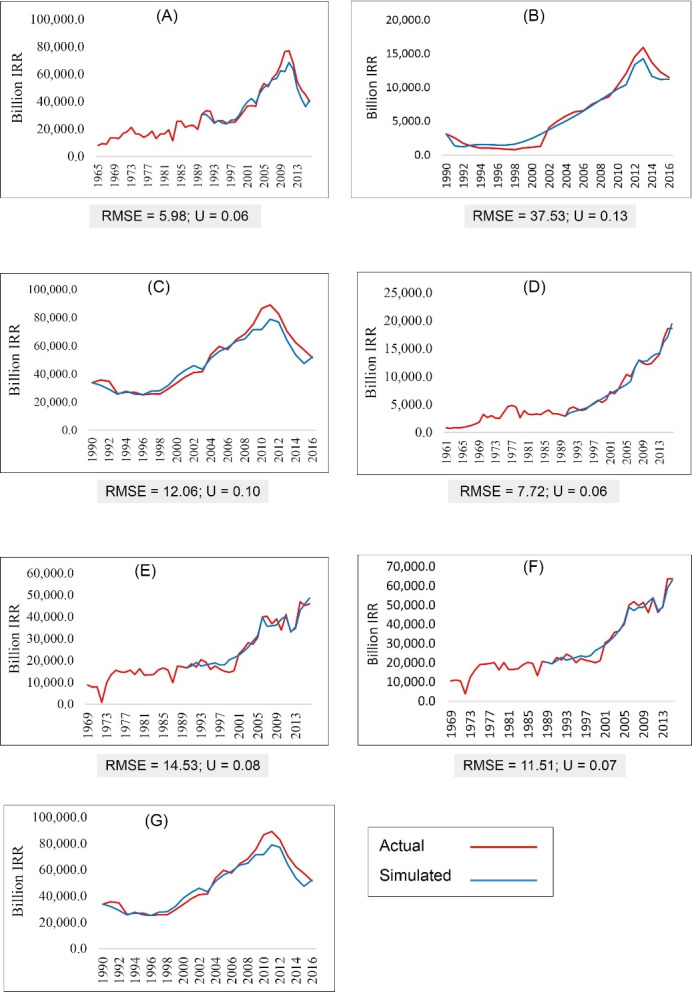
The Simulation of Iran’s Health Expenditure Variables in the Equations System: (A) Out-of-pocket health expenditure, (B) prepaid private health expenditure, (C) Private health expenditure, (D) Social Security Organization health expenditure, (E) government health expenditure, (F) Public health expenditure, (G) total health expenditure. Abbreviation: RMSE, root mean square error.

Figure 1 shows the simulation of HCE variables, the values of Utile indices (U), and the RMSE of their simulation. The model can simulate the trend of variables well. Utile indices and the RMSE for other simulated variables are presented in Table S44 (see Supplementary file 4).

 Table indicates the predictions of HCE variables, GDP, and the government’s revenues and expenditures. Hence, under the scenario of a 5% increase in oil revenues, it is predicted that the total HCE of 2030 will be 46 times larger than that of 2016. Indeed, it increased to 55 547 040 000.00 million rials in 2030 from 1 209 785 292.00 million rials in 2016. If the prediction is due to the fixed prices of 2004, then the THCE of the period will be 1.8 times larger and increase from 115 990 919.70 to 216 207 345.90 million rials.

**Figure 2:**
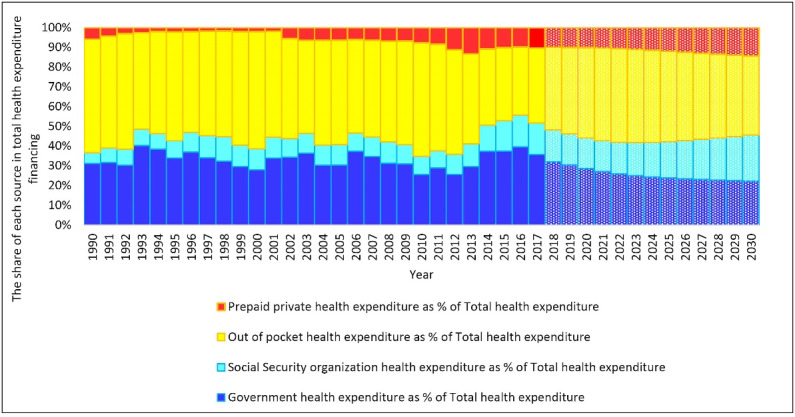
The Trend in Shares of Financing Factors in Iran’s Health Expenditure Coverage (1990-2030).

If the current situation continues, public healthcare costs are expected to grow steadily over the next few years, and the proportion of private healthcare costs is projected to remain higher than other sources of funding. Indeed, the share of public and private spending of total HCE will be 45.56% and 54.43%, respectively, in 2030. GHCE, the SOHCE, OOP, and PPHCE will cover 22.24%, 23.32%, 40%, and 14.53% of the THCE, respectively, in 2030 (Figure 2).

**Table:** The Health Expenditure Predicted for 2021 2026 and 2030

	**2016 (Million IRR)**		**2021(Million IRR)**		**2026 (Million IRR)**		**2030 (Million IRR)**		**Annualized Rate of Change (2016-2030)**
	**Current Price**	**Constant Price (2003)**	**Current Price**	**Constant Price (2003)**	**Current Price**	**Constant Price (2003)**	**Current Price**	**Constant Price (2003)**	
THCE	1 209 785 292.00	115 990 919.70	5 264 794 000.00	162 611 334.40	19 201 540 000.00	194 552 323.40	55 547 040 000.00	216 207 345.90	30.5
Public health expenditure	672 807 820.50	64 506 981.83	2 242 962 000.00	69 277 362.78	8 197 621 000.00	83 059 286.48	25 310 930 000.00	98 518 462.87	29.6
GHCE	478 761 811.50	45 902 378.86	1 424 433 000.00	43 995 824.14	4 485 271 000.00	45 445 307.72	12 354 780 000.00	48 088 866.54	25.9
SOHCE	194 046 009.00	18 604 602.97	818 528 600.00	25 281 526.29	3 712 350 000.00	37 613 978.76	12 956 140 000.00	50 429 557.41	34.4
Private health expenditure	536 977 471.50	51 483 937.83	3 021 832 000.00	93 333 971.66	11 003 920 000.00	111 493 047.00	30 236 110 000.00	117 688 883.00	33.5
OOP health expenditure	417 001 771.40	39 980 994.38	2 476 178 000.00	76 480 600.93	8 596 440 000.00	87 100 168.78	22 221 760 000.00	86 494 397.38	31.2
PPHCE	119 975 700.10	11 502 943.44	545 654 800.00	16 853 395.44	2 407 480 000.00	24 392 878.25	8 014 355 000.00	31 194 505.12	33.9

Abbreviations: THCE, total healthcare expenditure; GHCE, government health expenditure; SOHCE, social security organization health expenditure; OOP, out-of-pocket; PPHCE, prepaid private health expenditure.

**Figure 3:**
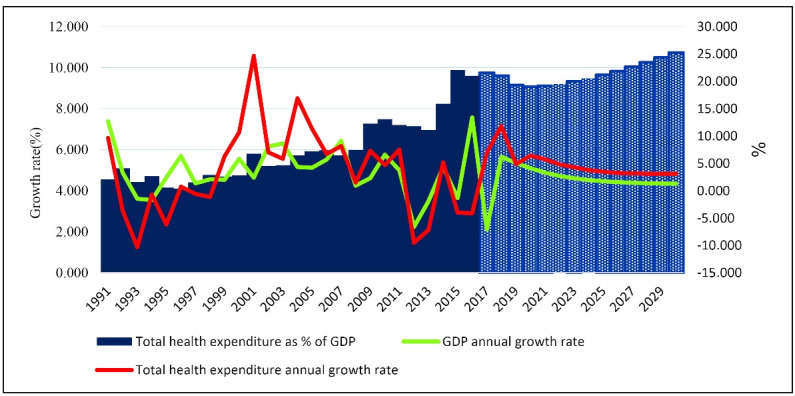
Previous Trend and Future Values of Health Expenditure as a Percentage of GDP (Right Side); Comparison of Health Expenditure Growth Rate With GDP Growth Rate (Left Side). Abbreviation: GDP, gross domestic product.

The average growth rate of THCE will be nearly 4% since 2020, greater than the average GDP growth rate, approximately 2%. The THCE to GDP ratio will experience a descending trend reaching 9% in 2020 from 9.6% in 2016. Then, with an increasing trend, it reaches 10.76% in 2030 (Figure 3).

**Figure 4:**
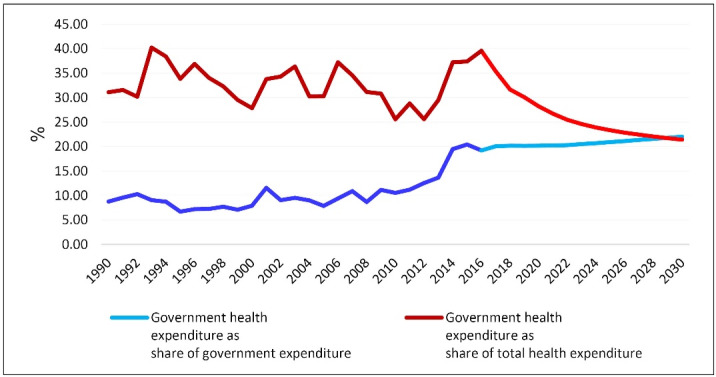
Iran’s Government Health Expenditure as a Percentage of Total Government Expenditure and Total Healthcare Expenditure.

Although the annual budget deficit will rise in the coming years, Iran’s government will cover a larger HCE share. The share of HCE from the total government’s expenditures will increase from nearly 19.2% in 2016 to 22% in 2030. However, since HCE grows faster than the government’s total expenditures, the ratio of GHCE to the THCE will gradually decrease and reach nearly 22.2% in 2030 from 35.5% in 2016 (Figure 4).

## Discussion

This study was conducted to project Iran’s HCE concerning macroeconomic conditions. Due to the results, if Iran’s oil revenues increase by 5%, THCE is 46 times greater in 2030 than in 2016 at current prices. Iran’s high inflation rate primarily causes this rapid growth. After adjusting for the inflation rate, HCE grows 1.8 times over the period by constant prices. Due to the results of Institute for Health Metrics and Evaluation study, Iran’s THCE (purchasing power parity $ 2017) will increase from $109 766 045 in 2016 to $183 223 156 in 2030. In other words, it becomes 1.66 times larger,^53^ a finding which is slightly different from the results of our study.

As mentioned earlier, there is a significant difference between the HCE of nominal prices and that of actual prices in Iran due to the high inflation rate. Inflation is a sustained increase in the general price of goods and services in an economy over time.^54^ Inflation sustainability is a fundamental feature of Iran’s economy.^55^ In inflation conditions, some population groups with fixed incomes lose their purchasing power and become poorer. The persistence of these conditions, especially in developing countries like Iran, will increase income inequality. In the health sector, this inequality will have many adverse effects, such as the increased chances of families encountering catastrophic health expenditures because of the nature of the market and – inelasticity of demand for healthcare.^56,57^ According to the Central Bank of the Islamic Republic of Iran, the health sector’s inflation is even higher than the general inflation.^57^ However, it should be noted that Iran is not the only country where inflation is higher in the healthcare sector than the general inflation, which is also true about other countries.^54,58^

Nowadays, developed countries encounter the challenge of an increased HCE to GDP ratio, which has increasingly been worrying policy-makers of such countries.^59^ Our results show that as a percentage of GDP, HCE increases from 9.6% in 2016 to 10.76% in 2030 because HCE grows faster than GDP. Over the past 15 years, the global health economy has experienced annual growth of 4% on average compared to the global economy with an average growth rate of 2.8%.^49^ Because of the increase in per-capita income and economic growth, people spend most of their income to increase welfare by spending in the service sector. Health is an integral part of these services, which are considered.^60^ The global HCE will be 8% of GDP on average in 2030. *This increase was predicted* to be from 6.1% to 12.6% (with an average of 8.8%) for Iran,^53^ consistent with our study findings. When HCE growth outpaces GDP growth, national economic sustainability may be weakened.^10^ Therefore, these findings can remind the policy-makers of the necessity of adopting specific approaches to curb HCE. Adopting appropriate policies to reduce the growth of HCE requires identifying major cost drivers first.^61^ The growth of HCE is caused by population growth, income per capita, inflation, and the excess growth of HCE due to the development of health technologies and increased expectations of patients. The excess growth is responsible for increased HCE as a percentage of GDP, which challenges financial sustainability, the primary cause of the increase in HCE observed in the US since the late 1950s. After a few decades, such a growth rate has started in many low- and middle-income countries such as Iran on a much smaller scale.^62^ Some of the main factors driving the growth of Iran’s HCE included an increase in income per capita, literacy rate, urbanization rate, and physicians.^63^ Some of the actions which were taken in Iran in recent decades to control costs and improve efficiency extend the primary healthcare network, develop a health expenditure monitoring system through the insurance mechanism, implement the family physician plan, establish the rural health insurance and referral system in rural areas and towns with fewer than 20 000 people, implement the hospital authority plan, merging some essential insurance funds into the Iran Health Insurance Organization,^64^ and establishing National Plan for Health Technology assessment^65^ However, there is a long way to go to achieve the desired results of these plans.

Although public funds are the cornerstone of sustainable financing for UHC in most countries,^66^ the predictions show that sharing the public spending in HCE financing slightly changes in future. In general, the shock caused by increased public expenditure due to the implementation of HTP, implemented in 2014, will wear off, and the public sector’s share of HCE financing will decrease. The government tries to play a more significant role in health system financing; therefore, GHCE as a percentage of total government expenditure increases from 19.2% to 22%. However, since HCE (31.49%) grows faster than government revenues (25.27%) and expenditures (24.9%), the share of GHCE from THCE decreases from 39.5% in 2016 to 22.24 in 2030. In recent years, the concept of fiscal space for health, whether the government can allocate additional resources to the health sector without prejudicing its financial sustainability, has grabbed much attention.^67^ The expansion of Fiscal space for health depends on favorable macro-economic environments, including sustained economic growth, high revenue mobilization capacity, and low levels of fiscal deficits.^22^ Using a macro-fiscal framework, our results imply that Iran’s government had limited fiscal space for health. According to the estimations, government expenditure will be higher than its revenues in the future, and Iran’s budget deficit will increase the following trends. Due to the International Monetary Fund, Iran’s budget deficit lasts until 2023.^68^ Based on a report published by the Research Center of Parliament, to increase economic sustainability, any budgetary commitment should be considered carefully in the future.^69^ Hence, financial sustainability should not be neglected to impose higher health expenditures on the government budget. The main drivers of fiscal space for the health sector can be classified into five categories: favorable macroeconomic conditions, reprioritizing the budget towards health, earmarked funds, development assistance for health,^49^ and efficiency gains.^70,71^ Considering several factors, including the decreasing trend of development assistance for health for the upper-middle-income countries,^34^ unsuitable macro-economic conditions of Iran, the limited potential to increase fiscal space for health through increasing health sector budget due to macroeconomic conditions, it seems that the best mid-term options to expend the fiscal space for health are cost management and efficiency improvement. Although several studies evaluated the capacity of earmarking in the expansion of fiscal space for health as moderate,^72^ there is insufficient evidence for judging the financial capacity of this option in Iran; thus, further research is needed.

Iran has had a health insurance system since 1947 of which has significantly developed. The SSO, Armed Forces Insurance, Iran’s Health Insurance, and Imam Khomeini Relief Foundation are now Iran’s major health insurance pools.^73^ In their study, Davari et al classified Iran’s health insurance system challenges as several categories, including increased growth of health expenditure, lack of systematic assessment of health technology, the limitations of financial resources, managerial challenges, regulation challenges, and the high number of uninsured individuals.^74^ Iran’s health insurance system could not achieve a universal insurance coverage in terms of specific reasons such as unknown insured rate, regressive financing, and non-transparent financial flow, fragmented and non-compulsory system, non-scientifically designed benefits package, non-health-oriented and expensive payment system; uncontrolled demands, and the administrative deficiency.^75^ Due to data limitations, it was impossible to simulate and project the expenditure growth of each health insurance scheme separately. Since Armed Forces Insurance, Iran’s Health Insurance, and the Imam Khomeini Relief Foundation receive a budget from the central government, their health expenditure growth rates were predicted under GHCE, and only SSO was estimated separately.

Due to the estimations, sharing SSO spending from THCE increases from 16.03% in 2016 to 23.32% in 2030. However, other studies show that SSO financial condition is not promising. For instance, the reports published by the Research Center of the Islamic parliament showed that SSO would face financial crises in the near future.^76^ If so, Iran’s government will lack the financial resources required to support; as a result, there is a concern that a considerable portion of the financial burden of health expenditure is imposed on the private sector, including households.

Sharing the private funds from THCE increases in future. The private expenditure consists of households (OOP), firms, non-profit organizations, private medical insurance schemes, and other private sector organizations which are collectively projected in the form of PPHCE. Due to the estimations, OOP as a percentage of THCE increased from 34.4% in 2016 to 40% in 2030. Projected OOP payments will be lower than those after the implementation of HTP; however, it exceeds OOP payments during the 2014-2016 period. Nevertheless, the predictions show that sharing the OOP payments of private spending decreases from 77.65% in 2016 to 73.49% in 2030, and the PPHCE of health financing will increase. The findings conducted by Institute for Health Metrics and Evaluation confirm the occurrence of this pattern in Iran’s health system.^53^ In 2016, nearly 58.6% of this item came from private medical insurance companies. The debate in the international health community on the role of private coverage has often been characterized by an easy dismissal of private insurance as fundamentally undesirable and destined to erode equity and efficiency in healthcare.^77^ The lack of an effective health financing system has become among the most critical issues, especially in underdeveloped countries. The high proportion of OOP payments is an ineffective health financing system. Private health insurance can support families by distributing health risks among many people and protecting them from incurring catastrophic health expenditures.^78^ The evidence shows that private insurance can play a positive role in promoting equity and accessibility in developing countries that are managed in a good manner.^79^ There is a wide range of tools and experiences to regulate the private health insurance market, using which this financing mechanism will be able to play a positive role in developing equitable health system. positive equitable health systems. Policy-makers should actively understand the value of these tools and employ them to serve the needs of the public.^77^

 THCE can be predicted from different perspectives. For instance, it can be predicted to focus on diseases, different age groups, services, and functions. However, this study analyzed the ability of each primary source of financing for healthcare which its results may be used as a basis for any health financing reform to accelerate achieving UHC. Hence, it seems that all financing sources should be considered to have a comprehensive evaluation of Iran’s health system’s future status. At the same time, future studies should focus on designing a sustainable system for health financing and increasing the system’s resilience against potential economic shocks to prevent the adverse effects of different crises such as economic sanctions and recessions on public health.

This study has several limitations which should be mentioned. At first, due to data limitations, it was impossible to project the changes in all sources of health financing in detail, so all financing sources were divided into four main groups (government, SSO, households, and other private agents), and forecasts are provided for these four major groups. Second, the necessary data were collected from various sources. Although the statistical caliber of these databases may be inconsistent, it should be noted that there are usually different institutions for data collection and reporting at the national and international levels, depending on the nature of a variable. Therefore, the diversity of databases is not unique to our study. For example, there has been this diversity in the study by Dieleman et al.^36^ It is hoped that improving databases such as National Health Accounts will provide a complete set of time-series data to perform more accurate evaluations in the future. Due to the unavailability of data, the simulation was conducted until 2016 (before the sanctions), and it was impossible to analyze the effect of the recent sanction shock. Fourth, despite the considerable role of oil and gas revenue in total government revenue, it was impossible to predict it due to the high complexity of the oil market worldwide. Therefore, oil revenue was considered exogenous in the developed model, assuming a 5% annual growth. Another limitation of this study is that it does not consider health outcome variables such as life expectancy, maternal mortality, infant mortality, non-communicable and other diseases burden. The explanatory variables of this study have often been economic and reflect the capacity of the Iranian economy to spend in the health system, but it does not reflect how much this expenditure should be given to the overall goals of the health sector. In some studies, the minimum sample size for ARDL is often considered to be 30 observations. In estimating most of the equations designed in this study, the sample size was more than 30 observations. For example, 58 observations were used in estimating the GDP equation. Due to data limitations on variables such as prepaid folk medical costs, its ARDL equation was estimated based on 26 observations, limiting the simulation of the entire macrostructure model based on 26 data points. The cost management and efficient use of existing resources should seriously be considered. It is also necessary to evaluate the government’s capacity to expand fiscal space for health in the mid-term so that the health financing system can move towards more sustainability. Since private health insurances are likely to grow, appropriate regulatory strategies should also be developed to control the health insurance market to take advantage of their capacities appropriately and improve the population’s health.

## Conclusion

 To conclude, due to our findings, until 2030, Iran’s HCE grows faster than GDP and government revenue and expenditure. Despite the increased proportion of Iran’s GHCE to the total government expenditure, it experiences a descending trend as a percentage of THCE; therefore, efforts to increase efficiency and control costs to match the growth rate of health expenditures with GDP and government financial capacity can be of significant importance in ensuring sustainable health financing. OOP payments will still be a significant source of THCE financing. Sharing PPHCE and SOHCE increases in health financing. Hence, the government and public and private insurance systems should make the reforms to guarantee access to necessary healthcare services of favorable quality, decrease OOP payments, and increase financial protection among households.

## Ethical issues

 Ethical approval for the research was received from the Shahid Beheshti University of Medical Sciences with approval code IR.SBMU.PHNS.REC.1396.80.

## Competing interests

 Authors declare that they have no competing interests.

## Authors’ contributions

 All authors have contributed to this study. Conception and design (NJ, SD, MN, RG); Acquisition of data (NJ, SD, MN, ZA); Analysis and interpretation of data (MN, NJ, SD); Drafting of the manuscript (SD, NJ, ZA, RG); Critical revision of the manuscript for important intellectual content (NJ, SD, MN, ZA, RG). All authors have read and approved the manuscript.

## Funding

 This work was supported by the National Institute for Health Research of Islamic Republic of Iran. This paper extracted from results of a research project entitled “Analysis of fiscal space for health in Iran and forecasting it for 2021, 2026 & 2030” supported by the National Institute for Health Research under contract/grant number 241/M/9658. The institute provided only financial support for the entire project and did not have any direct contribution in the design of the study, data collection, analysis, and preparation of this manuscript.

## Supplementary files


Supplementary file 1. Data Sources.

Supplementary file 2. Determining the Variables Cointegration Rank.

Supplementary file 3. Details of Econometric Models.

Supplementary file 4. Validity Assessment.
