# Supplementary material for: The Projection of Iran’s Healthcare Expenditures By 2030: Evidence of a Time-Series Analysis
Source: Int J Health Policy Manag. 2022 Feb 1;11(11):2563–73. doi: 10.34172/ijhpm.2022.5405 (PMC9818126; doi:10.34172/ijhpm.2022.5405)
Supplement: Supplementary file 1 — Data Sources. [file ijhpm-11-2563-s001.pdf]

Article title: The Projection of Iran's Healthcare Expenditures By 2030: Evidence of a Time-Series Analysis

Journal name: International Journal of Health Policy and Management (IJHPM)

Authors' information: Nader Jahanmehr[1], Mohammad Noferesti[2], Soheila Damiri[3]*, Zhaleh Abdi[4], Reza Goudarzi[5]

[1]Health Economics, Management and Policy Department, Virtual School of Medical Education & Management, Shahid Beheshti University of Medical Sciences. Tehran. Iran.

[2]Department of Economics, School of Economics and Political Sciences, Shahid Beheshti University, Tehran, Iran.

[3]Department of Health Management & Economics, School of Public Health, Tehran University of Medical Sciences, Tehran, Iran.

[4]National Institute of Health Research, Tehran University of Medical Sciences, Tehran, Iran.

[5]Health Services Management Research Center, Institute for Futures Studies in Health, Kerman University of Medical Sciences, Kerman, Iran.

(*Corresponding author: damiri.soheila@gmail.com)

Supplementary file 1. Data Sources

In this study, many variables have been used to achieve the intended goals. The required data has been collected from several national databases. Table 1 presents the variables extracted from each database.

Table S1: Data sources

Databases	Variables
Statistical Center of Iran<br>https://www.amar.org.ir	• Total Health Expenditure<br>• Out of Pocket Health Expenditure<br>• Private Health Expenditure<br>• Prepaid Private Health Expenditure<br>• Social Security Organization Health Expenditure<br>• Government Health Expenditure<br>• Foreign Health Expenditure
Central Bank of Iran<br>https://www.cbi.ir/	• Healthcare Consumer Price Index<br>• Gross Domestic Product<br>• Government Tax Revenue<br>• GDP deflator<br>• Public Health Insurance Coverage<br>• Public Health Expenditure<br>• Inflation rate<br>• Total labour force<br>• Oil and Gas revenue<br>• Government Revenue<br>• Capital accumulation of all sectors of the economy<br>• Real wage index<br>• Consumer Price Index<br>• Total investment<br>• Interest rates on long-term deposits<br>• Government Current Payments<br>• Liquidity<br>• Oil and Gas Exports(mollion $)
World Population Prospects: The 2017 Revision<br>https://esa.un.org/unpd/wpp/Download/Standard/Mortality/	• Total Population<br>• Urbanization rate<br>• Share of population > 60 years<br>• 20 years <Share of population< 60 years<br>• Active population
Statistical reports of the Social Security Organization<br>https://www.tamin.ir/News/Item/101014?catid=223	• Social Security Organization Insurance Coverage<br>• Social Security Organization Revenue
Statistical reports of the Central Insurance of Iran<br>https://www.centinsur.ir/en-US/Portal/1/page/Home	• Commercial Health Insuran Revenue
