# Supplementary material for: The Projection of Iran’s Healthcare Expenditures By 2030: Evidence of a Time-Series Analysis
Source: Int J Health Policy Manag. 2022 Feb 1;11(11):2563–73. doi: 10.34172/ijhpm.2022.5405 (PMC9818126; doi:10.34172/ijhpm.2022.5405)
Supplement: Supplementary file 2 — Determining the Variables Cointegration Rank. [file ijhpm-11-2563-s002.pdf]

Article title: The Projection of Iran's Healthcare Expenditures By 2030: Evidence of a Time-Series Analysis

Journal name: International Journal of Health Policy and Management (IJHPM)

Authors' information: Nader Jahanmehr[1], Mohammad Noferesti[2], Soheila Damiri[3]*, Zhaleh Abdi[4], Reza Goudarzi[5]

[1]Health Economics, Management and Policy Department, Virtual School of Medical Education & Management, Shahid Beheshti University of Medical Sciences. Tehran. Iran.

[2]Department of Economics, School of Economics and Political Sciences, Shahid Beheshti University, Tehran, Iran.

[3]Department of Health Management & Economics, School of Public Health, Tehran University of Medical Sciences, Tehran, Iran.

[4]National Institute of Health Research, Tehran University of Medical Sciences, Tehran, Iran.

[5]Health Services Management Research Center, Institute for Futures Studies in Health, Kerman University of Medical Sciences, Kerman, Iran.

(*Corresponding author: damiri.soheila@gmail.com)

Supplementary file 2. Determining the Variables Cointegration Rank

In time series studies, evaluating the stationarity of variables is one of the essential steps. In this study, the Augmented Dicky–Fuller (ADF) test was used to evaluate the stationarity of the variables. The results of this evaluation are presented in the table 2.

Table S2 Augmented Dicky–Fuller (ADF) test results

Variable	(C, T, P)*	P-value	Difference	(C, T, P)	P-value	Result
GDP	(-, -, 1)	0.001	-	-	-	I(0)
LOG(GDP)	(C, T, 1)	0.271	DLOG(GDP)	(C, -, 0)	0.005	I(1)
K	(-, T, 1)	0.625	D(K)	(-, -, 0)	0	I(2)
L	(C, -, 1)	0.044	D(L)			I(0)
LOG(L)	(C, -, 2)	0.332	DLOG(L)	(C, -, 0)	0	I(1)
W/CPI	(-, T, 1)	0.388	D(W/CPI)	(-, -, 1)	0	I(1)
F	(-, T, 9)	0.993	D(F)	(-, T, 4)	0.045	I(1)
POP2060	(-, -, 1)	0	-	-	-	I(0)

CPI	(-, -, 10)	1	D(CPI)	(C, T, 2)	0	I(1)
P	(-, T, 1)	0.995	DP	(-, T, 5)	0.46	I(1)
I	(-, T, 1)	0.268	D(I)	(-, -, 0)	0	I(1)
R	(C, -, 0)	0.437	D(R)	(-, -, 0)	0	I(1)
TAXJ/P	(-, -, 0)	0.976	D(TAXJ/P)	(-, -, 0)	0	I(1)
GEJ/P	(-, -, 1)	0.728	D(GEJ/P)	(-, -, 0)	0	I(1)
XORJ/P	(C, -, 0)	0.152	D(XORJ/P)	(-, -, 0)	0	I(1)
M2j	(-, -, 3)	0.998	DM2j	(-, -, 1)	0.0083	I(2)
HCPI	(-, -, 5)	0.376	D(HCPI)	(-, T, 1)	0	I(2)
GEJ	(-, -, 0)	1	D(GEJ)	(-, -, 0)	0	I(2)
XOG	(-, T, 0)	0.254	D(XOG)	(-, -, 0)	0	I(1)
GRJ/P	(-, -, 0)	0.642	D(GRJ/P)	(-, -, 0)	0	I(1)
LOG(OHEXJ/HCPI)	(-, T, 0)	0.66	D(OHEXJ/HCPI)	(-, -, 0)	0	I(1)
LOG(GDP-(TAXJ/P))	(C, T, 1)	0.136	DLOG(GDP-(TAXJ/P))	(-, -, 0)	0.003	I(1)
LOG(PUBHEXJ/HCPI)	(C, T, 0)	0.013	-	-	-	I(0)
ICOV	(-, -, 0)	0.992	D(ICOV)	(-, -, 0)	0.003	I(1)
LOG(ICOV)	(-, -, 0)	0.989	DLOG(ICOV)	(C, T, 0)	0.004	I(1)
POPT	(C, -, 1)	0.001	-	-	-	I(0)
PPHEXJ/HCPI	(C, T, 1)	0.238	D(PPHEXJ/HCPI)	(-, -, 0)	0.01	I(1)
LOG(POPT)	(C, -, 3)	0.012	-	-	-	I(0)
PIRJ/P	(-, -, 0)	0.999	D(PIRJ/P)	(C, T, 0)	0	I(1)
PPHEXJ/HCPI	(C, T, 1)	0.238	D(PPHEXJ/HCPI)	(-, -, 0)	0.01	I(1)
LOG(PIR/P)	(C, -, 0)	0.023	-	-	-	I(0)
GHEXJ/HCPI	(C, T, 0)	0.316	D(GHEXJ/HCPI)	(-, -,0)	0	I(1)
POP15R	(C, T, 1)	0.001	-	-	-	I(0)
URR	(C, -, 1)	0.102	D(URR)	(-, -, 0)	0	I(2)
SHIJ/HCPI	(-, -, 0)	1	D(SHIJ/HCPI)	(-, T, 0)	0	I(1)
SIRJ/P	(-, -, 4)	1	D(IRJ/P)	(-, -, 1)	0	I(2)
SICOV	(-, -, 1)	0.649	D(SICOV)	(C, -, 0)	0.053	I(1)
POP60R	(C, -, 1)	1	D(POP60R)	(-, -, 0)	0	I(2)

*C: Intercept, t: Trend and P number of lags
