# Supplementary material for: The Projection of Iran’s Healthcare Expenditures By 2030: Evidence of a Time-Series Analysis
Source: Int J Health Policy Manag. 2022 Feb 1;11(11):2563–73. doi: 10.34172/ijhpm.2022.5405 (PMC9818126; doi:10.34172/ijhpm.2022.5405)
Supplement: Supplementary file 3 — Details of Econometric Models. [file ijhpm-11-2563-s003.pdf]

Article title: The Projection of Iran's Healthcare Expenditures By 2030: Evidence of a Time-Series Analysis

Journal name: International Journal of Health Policy and Management (IJHPM)

Authors' information: Nader Jahanmehr[1], Mohammad Noferesti[2], Soheila Damiri[3]*, Zhaleh Abdi[4], Reza Goudarzi[5]

[1]Health Economics, Management and Policy Department, Virtual School of Medical Education & Management, Shahid Beheshti University of Medical Sciences. Tehran. Iran.

[2]Department of Economics, School of Economics and Political Sciences, Shahid Beheshti University, Tehran, Iran.

[3]Department of Health Management & Economics, School of Public Health, Tehran University of Medical Sciences, Tehran, Iran.

[4]National Institute of Health Research, Tehran University of Medical Sciences, Tehran, Iran.

[5]Health Services Management Research Center, Institute for Futures Studies in Health, Kerman University of Medical Sciences, Kerman, Iran.

(*Corresponding author: damiri.soheila@gmail.com)

Supplementary file 3. Details of Econometric Models

All characteristics of the final models that have been specified and included in the final macro-structural econometric model.

Table S3: ARDL nodel for GDP (Gross Domestic Product)

Dependent Variable	Gross Domestic Product(GDP)
Type of regression equation	ARDL(1, 0, 1)
dynamic equation	LOG(GDP) = -1.357864 + 0.486*LOG(GDP(-1)) + 0.293*LOG(L) + 2.639*LOG(K)<br>(0.416)[1] (0.057) (0.040) (0.319)<br>+ 2.40*LOG(K(-1)) + 0.164*D4753 – 0.147*D5960<br>(0.288) (0.023) (0.031)
R- squared	0.994

[1] These numbers indicate the standard deviation of the estimated coefficients.

Banerjee, Dolado, and Master test	Banerjee St= (0.86-1)/(0.057) = - 9.01<br>Critical St = -3.91 Result: The existence of a long-run relationship is confirmed
long run equation	GDP = -2.645 + 0.572*LOG(L) + 0.462*LOG(K) + 0.320*D4753 – 0.288*D5960<br>(0.847) (0.078) (0.034) (0.038) (0.068)
Short run equation	DLOG(GDP) = 0.293*DLOG(L) + 2.639*DLOG(K) + 0.164*D(D4753) – 0.147*D(D5960)<br>(0.040) (0.319) (0.023) (0.031)<br>- 0.513*CointEQ<br>(0.057)

Table S4: ARDL nodel for Total labour force

Dependent Variable	Total labour force
Type of regression equation	ARDL(1, 1, 0)
dynamic equation	LOG(L) = 0.177 + 0.449*LOG(L(-1)) + 0.044*LOG(W/CPI) – 0.035*LOG(W(-1)/CPI(-1)) +<br>(0.104) (0.066) (0.013) (0.012)<br>+ 0.536*LOG(F)<br>(0.065)
R- squared	0.999
Banerjee, Dolado, and Master test	Banerjee St= ((0.449)-1)/(0.066) = - 8.34<br>Critical St = -3.91 Result: The existence of a long-run relationship is confirmed
long run equation	LOG(L) = 0.323 + 0.015*LOG(W/CPI) + 0.974*LOG(F)<br>(0.191) (0.005) (0.011)
Short run equation	DLOG(L) = 0.044*DLOG(W/CPI) + 0.536*DLOG(F)) – 0.716*CointEQ(-1)<br>(0.013) (0.065) (0.066)

Table S5: ARDL nodel for Active population

Dependent Variable	Active population
Type of regression equation	ARDL(1, 0)
dynamic equation	LOG(F) = 0.875 +0.733*LOG(F(-1)) + 0.208*LOG(POP2060) – 0.043*D9093+ 0.050*D9495<br>(0.279) (0.077) (0.059) (0.007)
R- squared	0.999
Banerjee, Dolado, and Master test	Banerjee St= ((0.083)-1)/(0.021) = - 3.46<br>Critical St = - 3.28 Result: The existence of a long-run relationship is confirmed
long run equation	LOG(F) = 3.288 +0.783*LOG(POP2060) – 0.162*D9093<br>(0.267) (0.016) (0.042)
Short run equation	DLOG(F) = 0.208*DLOG(POP2060) – 0.043*D(D9093) – 0.266* CointEQ<br>(0.059) (0.007) (0.077)

Table S6: OLS nodel for Consumer Price Index

Dependent Variable	Consumer Price Index
Type of regression equation	OLS
dynamic equation	CPI = 0.274935613587*P + 0.850610900925*CPI(-1) + 0.39346369618*D9192
R- squared	0.999

Table S7: ARDL nodel for Total investment

Dependent Variable	Total investment
Type of regression equation	ARDL(1, 0, 0)
dynamic equation	I = -24600.14 + 0.272*I(-1) + 0.298*GDP – 5531.063*R -56700.71*D4753 – 87779.76*D9194<br>(11708.38) (0.083) (0.034) (1511.55) (15476.3) (15476.3)
R- squared	*0.973*
Banerjee, Dolado, and Master test	Banerjee St= ((0.272)-1)/(0.083) = - 8.77<br>Critical St = - 3.57 Result: The existence of a long-run relationship is confirmed
long run equation	I= -33822.2 + 0.409*GDP – 7604.53*R–77956.59*D4753 – 120686.5*D9194<br>(16202.8) (0.019) (1890.7) (18050.6)
Short run equation	D(I) = 0.298*D(GDP) -5531.06*D(R) -56700.7*D(D4753) – 87779.76*D(D9194)<br>(0.034) (1511.55) (15476.3) (18353.55)<br>0.727*CointEQ<br>(0.083)

Capital accumulation of all sectors of the economy is specified as a definitional equation in the final model. The variables of this equation include total investment and the negative effect of the destruction of capital caused by the imposed Iraq war and Rudbar earthquake. The coefficients of this definitional equation are extracted from the reports of the Plan and Budget Organization and the Ministry of Economic Affairs and Finance. These coefficients have been used in other studies[1].

Table S8: Specified equation for Capital accumulation of all sectors of the economy

Dependent Variable	Capital accumulation of all sectors of the economy
Type of regression equation	OLS
dynamic equation	k = 0.955 * k(-1) - 0.378527 * wd - 0.153215 * erd + i
R- squared	

Table S9: ARDL nodel for Tax Revenue

Dependent Variable	Tax Revenue
Type of regression equation	ARDL(1, 1)
dynamic equation	(TAXJ/P) = -85.858 + 0.651*(TAXJ(-1)/P(-1)) + 0.064*GDP -0.043*D9495<br>(3964.57) (0.117) (0.019) (8723.04)

R- squared	0.918
Banerjee, Dolado, and Master test	Banerjee St= ((0.651)-1)/(0.117) = - 3.172<br>Critical St = - -2.93 [2]Result: The existence of a long-run relationship is confirmed
long run equation	D(TAXJ/P) = -246.38 + 0.059*GDP + 99999.77*D9495<br>(0.125) (0.000) (7649.77) (5770.41)
Short run equation	(TAXJ/P) = 0.064*D(GDP) + 34847.4*D(D9495) – 0.348* *CointEQ(-1)<br>(0.018) (8723.04) (0.117)

Table S10 ARDL nodel for Government Current Payments

Dependent Variable	Government expenditure
Type of regression equation	ARDL(1, 0, 0)
dynamic equation	(GEJ/P) = 14843.48 + 0.438*(GEJ(-1)/P(-1)) + 0.202*(XORJ/P) + 0.763*(TAXJ/P) -<br>(5118.02) (0.063) (0.029) (0.082)<br>21683.07*D8992<br>(7153.19)
R- squared	*0.958*
Banerjee, Dolado, and Master test	Banerjee St= ((0.438)-1)/(0.063) = - 15.17<br>Critical St = - -3.57 Result: The existence of a long-run relationship is confirmed
long run equation	(GEJ/P) = 26443.5 + 0.361*(XORJ/P) + 1.360*(TAXJ/P) – 38628.17*D8992<br>(7887.8) (0.039) (0.104) (13747.7)
Short run equation	D(GEJ/P) = 0.202*D(XORJ/P) + 0.763*(D(TAXJ/P) -21683.07*D(D8992) –0.561*CointEQ(-1)<br>(0.029) (0.082) (7153.19) (0.063)

Table S11: ARDL nodel for Total government expenditure

Dependent Variable	**Total government expenditure**
Type of regression equation	ARDL(1, 1, 1)
dynamic equation	(GEPJ/P) = 12032.7 + 0.545*(GEPJ(-1)/P(-1)) + 1.521*(TAXJ/P) -0.883*(TAXJ(-1)/P(-1))<br>(7675.9) (0.092) (0.215) (0.312)<br>+0.811*(XORJ/P) – 0.315*(GRJ(-1)/P(-1)) + 66385*D85 + 719717.6*D87<br>(0.058) (0.110) (20266.3) (20449.8)
R- squared	*0.975*
Banerjee, Dolado, and Master test	Banerjee St= ((0.545)-1)/(0.092) = - 4.94<br>Critical St = - -3.57 Result: The existence of a long-run relationship is confirmed
long run equation	(GEPJ/P) = 26485.6 + 1.402*(TAXJ/P) + 1.092*(XORJ/P) + 146122.2*D85 + 158300*D87

[2] $\alpha = 0.10$

Short run equation	(16198) (0.217) (0.084) (53312) (52902.13)<br>D(GEPJ/P) = 1.521*D(TAXJ/P) + 0.811*D(XORJ/P) + 66385*D(D85) + 71917.6*D(D87)<br>(0.215) (0.058) (20266.3) (20449.83)<br>– 0.454*CointEQ(-1)<br>(0.092)

Table S12: ARDL nodel for Government Revenue

Dependent Variable	Government Revenue
Type of regression equation	**ARDL(1, 2, 1)**
dynamic equation	(GRJ/P) = -2225.99 + 0.587*(GRJ(-1)/P(-1)) + 1.509*(TAXJ/P) -1.018*(TAXJ(-1)/P(-1))+<br>(3219.78) (0.123) (0.096) (0.226)<br>1.164*(TAXJ(-2)/P(-2))+0.987*(XORJ/P) – 0.596*(GRJ(-1)/P(-1)) -10389.64*D7982<br>(0.097) (0.024) (0.121) (4239.89)
R- squared	***0.994***
Banerjee, Dolado, and Master test	Banerjee St= ((0.587)-1)/(0.123) = - 3.35<br>Critical St = - -3.20 [3] Result: The existence of a long-run relationship is confirmed
long run equation	(GRJ/P) = -5395 + 1.587*(TAXJ/P) + 0.948*(XORJ/P) – 25180.73*D7982<br>(7629.7) (0.100) (0.036) (13446.45)
Short run equation	D(GRJ/P) = 1.509*D(TAXJ/P) -0.164*(TAXJ(-1)/P(-1))+ 0.987*D(XORJ/P) –<br>(0.096) (0.097) (0.024)<br>10389.6*D(D7982) – 0.412*CointEQ(-1)<br>(4239.89) (0.123)

Table S13: ARDL nodel for GDP deflator

Dependent Variable	**GDP deflator**
Type of regression equation	ARDL(1, 0, 1)
dynamic equation	LOG(P) = 0.841 + 0.826*LOG(P(-1)) + 1.016*LOG(M2J) – 0.84*LOG(M2J(-1)) -<br>(0.645) (0.042) (0.171) (0.157)<br>0.436*LOG(GDP) + 0.464*LOG(GDP(-1)) – 0.256*LOG(GDP(-2)) – 0.258*D9495<br>(0.145) (0.234) (0.158) (0.053)
R- squared	0.999
Banerjee, Dolado, and Master test	Banerjee St= ((0.826)-1)/(0.042) = -4.14<br>Critical St = -3.57 Result: The existence of a long-run relationship is confirmed

[3] $\alpha = 0.10$

long run equation	LOG(P) = 4.842 + 1.006*LOG(M2J) – 1.31*LOG(GDP) – 1.486*D9495<br>(3.416) (0.038) (0.279) (0.443)
Short run equation	DLOG(P) = 1.016*DLOG(M2J) – 0.436*DLOG(GDP) + 0.256*DLOG(GDP(-1)) -<br>(0.171) (0.145) (0.158)<br>0.258*D(D9495)-0.173*CointEQ(-1)<br>(0.053) (0.042)

Table S14: ARDL nodel for Healthcare Consumer Price Index

Dependent Variable	**Healthcare Consumer Price Index**
Type of regression equation	**ARDL(1, 1, 1)**
dynamic equation	LOG(HCPI) = 0.077 + 0.89*LOG(HCPI(-1)) + 0.73*LOG(M2J) – 0.626*LOG(M2J(-1))<br>(0.66) (0.03) (0.181) (0.176)<br>-0.395*LOG(GDP) + 0.293*LOG(GDP(-1))<br>(0.145) (0.155)
R- squared	**0.999**
Banerjee, Dolado, and Master test	Banerjee St= ((0.896)-1/(0.03) = - 3.66<br>Critical St = -3.57 Result: The existence of a long-run relationship is confirmed
long run equation	LOG(HCPI) = 0.756 + 1.01*LOG(M2J) – 0.987*LOG(GDP)<br>(0.137) (0.036) (0.074)
Short run equation	DLOG(HCPI) = 0.730*DLOG(M2J) – 0.395*DLOG(GDP) -0.103* CointEQ(-1)<br>(0.181) (0.145) (0.038)

Table S15: ARDL nodel for Liquidity

Dependent Variable	**Liquidity**
Type of regression equation	**ARDL(1, 0, 1)**
dynamic equation	LOG(M2J) = -0.075 + 0.903*LOG(M2J(-1)) + 0.03*LOG(XOG) + 0.26*LOG(GEJ(-1))<br>(0.047) (0.021) (0.045) (0.051)<br>-0.156*LOG(GEJ(-1)) + 0.1*D58 -0.069*D8790<br>(0.048) (0.025)
R- squared	0.999
Banerjee, Dolado, and Master test	Banerjee St= ((0.903)-1/(0.021) = - 4.61<br>Critical St = -3.57 Result: The existence of a long-run relationship is confirmed
long run equation	LOG(M2J) = -0.786 + 0.319*LOG(XOG) + 1.081*LOG(GEJ) + 1.049*D85 – 0.720*D8790<br>(0.535) (0.112) (0.041) (0.571) (0.306)
Short run equation	DLOG(M2J) = 0.03*DLOG(XOG) + 0.260*DLOG(GEJ) + 0.109*D(D85) – 0.069*D(D8790) -<br>(0.007) (0.045) (0.048) (0.025)

	0.096* CointEQ(-1)<br>(0.021)

Table S16: ARDL nodel for Out of Pocket Health Expenditure

Dependent Variable	**Out of Pocket Health Expenditure**
Type of regression equation	ARDL(1, 1, 2, 0, 2)
dynamic equation	LOG(OHEXJ/HCPI) =2.628+0.129*LOG(OHEXJ(-1)/HCPI(-1)) +1.222*LOG(GDP-(TAXJ/P))<br>(0.935) (0.139) (0.405)<br>+0.732*LOG(GDP(-1)-(TAXJ(-1)/P(-1)) – 1.311*HCPI0 – 0.029*HCPI0(-1)-0.928*HCPI0(-2)<br>(0.407) (0.246) (0.151) (0.167)<br>-0.354*LOG(PUBHEXJ/HCPI) – 0.471*LOG(ICOV) – 0.186*LOG(ICOV(-1)) +<br>(0.092) (0.142) (0.159)<br>0.189*LOG(ICOV(-2)) + 0.288*D73 – 0.166*D82<br>(0.125) (0.068) (0.054)
R- squared	*0.983*
Banerjee, Dolado, and Master test	Banerjee St= ((0.129)-1)/(0.139) = -6.26<br>Critical St = -4.18 Result: The existence of a long-run relationship is confirmed
long run equation	LOG(OHEXJ/HCPI) =3.02 + 2.247*LOG(GDP-(TAXJ/P)) – 2.608*HCPI0 –<br>(1.135) (0.212) (0.375)<br>0.407*(PUBHEXJ/HCPI) -0.538*LOG(ICOV) +0.331*D73 – 0.191*D82<br>(0.115) (0.083) (0.094) (0.068)
Short run equation	DLOG(OHEXJ/HCPI) =1.22*DLOG(GDP-(TAXJ/P)) – 1.311*D(HCPI0)+0.928*D(HCPI0(-1))<br>(0.405) (0.246) (0.167)<br>-0.354*DLOG(PUBHEXJ/HCPI) – 0.471*DLOG(ICOV) – 0.189*DLOG(ICOV(-1)) +<br>(0.092) (0.142) (0.125)<br>0.288*D(D73) – 0.166*D(D82) – 0.87*Coint<br>EQ(-1)<br>(0.068) (0.054) (0.139)

Table S17: ARDL nodel for Public Health Insurance Coverage

Dependent Variable	**Public Health Insurance Coverage**
Type of regression equation	**ARDL(1, 0, 2)**
dynamic equation	ICOV =-(1.38E+08) + 0.418*ICOV(-1) + 3.04*POPT + 1.946*GDP – 1.485*GDP(-1)<br>(29649981) (0.117) (0.657) (8.425) (12.293)<br>-25.59*GDP(-2) + 7716326*D7677<br>(9.81) (1908140)
R- squared	***0.985***

Banerjee, Dolado, and Master test	**Banerjee St= ((0.418)-1)/(0.117) = -4.97**<br>**Critical: -3.57** **Result: The existence of a long-run relationship is confirmed**
long run equation	ICOV= -2341142 + 5.222*POPT – 43.22*GDP + 13271372*D7677<br>(20785053) (0.483) (8.252) (4554389.5)
Short run equation	D(ICOV)= 3.04*D(POPT) + 1.946*D(GDP) + 25.59*D(GDP(-1)) +7716325*D7677<br>(0.657) (8.425) (9.810) (1908139.8)<br>- 0.581*CointEQ(-1)<br>(0.117)

Table S18: ARDL nodel for Prepaid Private Health Expenditure

Dependent Variable	Prepaid Private Health Expenditure
Type of regression equation	ARDL(1, 0, 1, 1)
dynamic equation	(PPHEXJ/HCP) = 0.383*(PPHEXJ(-1)/HCPI(-1)) + 7.845*GDP -0.047*(PIRJ/P) +<br>(0.096) (1.376) (0.142)<br>0.384*(PIRJ(-1)/P(-1) – 3.315*POPT + 3.08*POPT(-1) + 3143117*D9192<br>(0.226) (2.220) (2.173) (456773.3)
R- squared	*0.990*
Banerjee, Dolado, and Master test	Banerjee St= ((0.383)-1)/(0.096) = -6.42<br>Critical: -3.91 Result: The existence of a long-run relationship is confirmed
long run equation	(PPHEXJ/HCPI)= 13.736*GDP + 0.547*(PIRJ/P) – 0.38*POPT + 5102305*D9192<br>(1.45) (0.200) (0.116) (1023789.8)
Short run equation	D(PPHEXJ/HCPI)= 7.845*D(GDP) -0.047*D(PIRJ/P) -3.315*D(POPT) + 3143116*D(D9192)<br>(1.37) (0.142) (2.220) (456773.3)<br>- 0.616*CointEQ(-1)<br>(0.096)

Table S19: ARDL nodel for Commercial Health Insuran Revenue

Dependent Variable	Commercial Health Insuran Revenue
Type of regression equation	ARDL(1, 2)
dynamic equation	LOG(PIRJ/P) = -6.472 + 0.769*LOG(PIRJ(-1)/P(-1)) – 0.371*LOG(GDP)<br>(4.836) (0.061) (1.441)<br>– 2.323*LOG(GDP(-1)) + 3.4*LOG(GDP(-2))<br>(2.281) (1.413)
R- squared	*0.0.977*
Banerjee, Dolado, and Master test	Banerjee St= ((0.769)-1)/(0.061) = -3.786<br>Critical: -3.35 Result: The existence of a long-run relationship is confirmed

long run equation	LOG(PIRJ/P)= -28.131 + 3.068*LOG(GDP)<br>(15.31) (1.056)
Short run equation	DLOG(PIRJ/P) = --0.371*DLOG(GDP) -3.4*DLOG(GDP(-1)) -0.23* CointEQ(-1)<br>(1.441) (1.413) (0.061)

Private health expenditures equation was specifed as a identity or definitional equation This equation is calculated as the sum of out-of-pocket health expenditure and prepaid private health expenditure.

phexj / hcpi = ohexj / hcpi + pphexj / hcpi

Table S20: ARDL nodel for Government Health Expenditure

Dependent Variable	Government Health Expenditure
Type of regression equation	ARDL(1, 0, 1, 2)
dynamic equation	(GHEXJ/HCPI) = 30839592 + 0.145*(GHEXJ(-1)/HCPI(-1)) + 19.26*(GRJ/P)<br>(15951117) (0.129) (6.255)<br>+(2.65E +08)*POP15R–(3.26E+08)*POP15R(-1)+(2.63E+08)*URR– (1.83E+0.9)*URR(-1)<br>(1.55E+08)) (1.62E +08) (5.96E+08) (1.09E +09)<br>+(1.60E+09)*URR(-2) – 6426336*D9192 + 6647172*D85<br>(6.25E+08) (2346855) (2812242)
R- squared	*0.952*
Banerjee, Dolado, and Master test	Banerjee St= ((0.145)-1)/(0.129) = -6.62<br>Critical: -3.82 Result: The existence of a long-run relationship is confirmed
long run equation	(GHEXJ/HCPI) = 36080275 + 22.535*(GRJ/P) – 7163566*POP15R + 33776597*URR<br>(18197722) (7.366) (22417105) (17356460)<br>-7518386*D9192 + 7776750*D85<br>(2779547.2) (3548219.8)
Short run equation	D(GHEXJ/HCPI) = 19262*D(GRJ/P) + 26479364*D(POP15R) + 26286092*D(URR)<br>(6.256) (154647486) (595739510)<br>-1596993*D(URR(-1)) – 6426336*D(D9192) + 6647172*D(D85) – 0.854**CointEQ(-1)<br>(624944238) (234685.9) (2812241.6) (0.129)

Table S21: ARDL nodel for Social Security Organization Health Expenditure

Dependent Variable	Social Security Organization Health Expenditure
Type of regression equation	ARDL(1, 2, 2, 0)
dynamic equation	(SHIJ/HCPI) = -2265926 + 0.371*(SHIJ(-1)/HCPI(-1))+0.048*(SIRJ/P) – 0.027*(SIRJ(-1)/P(-1)<br>(1419754) (0.101) (0.015) (0.026)

	+0.036*(SIRJ(-2)/P(-2)) – 0.046*SICOV – 0.305*SICOV(-1) + 0.404*SICOV(-2) +<br>(0.019) (0.154) (0.290) (0.176)<br>69655552*POP60R + 1211763*D5456 + 1613306*D8687<br>(30579493) (339164.7) (448541.4)
R- squared	*0.988*
Banerjee, Dolado, and Master test	Banerjee St= ((0.371)-1)/(0.101) = -6.227<br>Critical: -3.82 Result: The existence of a long-run relationship is confirmed
long run equation	(SHIJ/HCPI) = -3607112 + 0.091*(SIRJ/P) + 0.083*SICOV + 11088415*POP60R +<br>(2413274) (0.011) (0.031) (52254709)<br>1928997*D5456 + 2568211*D8687<br>(556008.5) (718771.7)
Short run equation	D(SHIJ/HCPI) = 0.048*D(SIRJ/P) – 0.036*D(SIRJ(-1)/P(-1) – 0.046*D(SICOV) –<br>(0.015) (0.019) (0.154)<br>0.404*D(SICOV(-1)) + 69655522*D(POP60R) + 1211763*D(D5456) + 1613306*D(D8687)<br>(0.176) (30579493) (339164.68) (448541.36)<br>- 0.628*CointEQ(-1)<br>(0.101)

Table S22: ARDL nodel for Social Security Organization Insurance Coverage

Dependent Variable	Social Security Organization Insurance Coverage
Type of regression equation	ARDL(1, 2, 0, 0)
dynamic equation	LOG(SICOV)=-6.451+ 0.735*LOG(SICOV(-1))+ 6.841*LOG(POPT) -15.479*LOG(POPT(-1))<br>(1.423) (0.046) (5.97) (12.01)<br>+9.779*LOG(POPT(-2)) + 0.187*LOG(GDP) – 0.729*LOG(L) – 0.185*D65<br>(6.187) (0.033) (0.108) (0.026)
R- squared	*0.998*
	Banerjee St= ((0.735)-1)/(0.046) = - 5.76<br>Critical: -3.57 Result: The existence of a long-run relationship is confirmed
long run equation	LOG(SICOV) = -24.349 + 4.309*LOG(POPT) + 0.708*LOG(GDP) – 2.752*LOG(L)<br>(1.829) (0.406) (0.134) (0.505)<br>-0.698*D65<br>(3161606.6)
Short run equation	DLOG(SICOV) = 6.841*DLOG(POPT) – 9.779*DLOG(POPT(-1)) + 0.187*DLOG(GDP)<br>(5.978) (6.187) (0.033)<br>-0.729*DLOG(L) – 0.185*D(D65) - 0.264*CointEQ(-1)

	(0.108) (0.026) (0.046)

Table S23: ARDL nodel for Social Security Organization Revenue

Dependent Variable	Social Security Organization Revenue
Type of regression equation	ARDL(1, 0, 0, 0)
dynamic equation	LOG(SIRJ/P) = -9.264 + 0.522*LOG(SIRJ(-1)/P(-1)) + 0.887*LOG(GDP)<br>(1.922) (0.083) (0.203)<br>+ 0.39*LOG(L) -0.124*LOG(XORJ/P) + 0.558*D5960 + 0.284*D9395<br>(0.183) (0.054) (0.112) (0.112)
R- squared	*0.976*
Banerjee, Dolado, and Master test	Banerjee St= ((0.522)-1)/(0.083) = - 5.75<br>Critical: -3.82 Result: The existence of a long-run relationship is confirmed
long run equation	LOG(SIRJ/P) =-19.40 + 1.858*LOG(GDP) + 0.817*LOG(L) – 0.26*LOG(XORJ/P)<br>(2.267) (0.383) (0.325) (0.109)<br>+1.17*D5960 + 0.596*D9395<br>(0.263) (0.23)
Short run equation	DLOG(SIRJ/P) = 0.887*DLOG(GDP) + 0.39*DLOG(L) – 0.124*DLOG(XORJ/P)<br>(0.203) (0.183) (0.054)<br>+ 0.558*D(D5960) + 0.284*D(D9395) – 0.477*CointEQ(-1)<br>(0.112) (0.112) (0.083)

Public health expenditures equation was specifed as a identity or definitional equation This equation is calculated as the sum of government health expenditure and Social Security Organization health expenditure.

pubhexj / hcpi = ghexj / hcpi + shij / hcpi

Finally, total health expenditures have been calculated using the following equation as the sum of public health expenditures and private health expenditures.

thexj / hcpi = pubhexj / hcpi + phexj / hcpi
