# Supplementary material for: The Projection of Iran’s Healthcare Expenditures By 2030: Evidence of a Time-Series Analysis
Source: Int J Health Policy Manag. 2022 Feb 1;11(11):2563–73. doi: 10.34172/ijhpm.2022.5405 (PMC9818126; doi:10.34172/ijhpm.2022.5405)
Supplement: Supplementary file 4 — Validity Assessment. [file ijhpm-11-2563-s004.pdf]

Article title: The Projection of Iran's Healthcare Expenditures By 2030: Evidence of a Time-Series Analysis

Journal name: International Journal of Health Policy and Management (IJHPM)

Authors' information: Nader Jahanmehr[1], Mohammad Noferesti[2], Soheila Damiri[3]*, Zhaleh Abdi[4], Reza Goudarzi[5]

[1]Health Economics, Management and Policy Department, Virtual School of Medical Education & Management, Shahid Beheshti University of Medical Sciences. Tehran. Iran.

[2]Department of Economics, School of Economics and Political Sciences, Shahid Beheshti University, Tehran, Iran.

[3]Department of Health Management & Economics, School of Public Health, Tehran University of Medical Sciences, Tehran, Iran.

[4]National Institute of Health Research, Tehran University of Medical Sciences, Tehran, Iran.

[5]Health Services Management Research Center, Institute for Futures Studies in Health, Kerman University of Medical Sciences, Kerman, Iran.

(*Corresponding author: damiri.soheila@gmail.com)

Supplementary file 4. Validity Assessment

Validity assessment

In this study, the credit assessment process is performed in two stages:

1. Evaluating the validity of the initial equations
2. Evaluating the validity of the final dynamic macro-structural dynamic econometric model

1. Evaluating the validity of the initial equations

Several equations were specified to estimate each of the dependent variables that had to be included in the dynamic macro-structural econometric model, but these equations had to address some regression. Each equation was repeated several times to finally we achieve a specification for each dependent variable equation that would both address these assumptions and simultaneously represent an appropriate simulation in the final macro-structural model. In this step four tests of Jarque–Bera, Breusch–Godfrey, White and Ramsey tests were utilized to check the normality of error terms distribution, lack of serial correlation between error terms and other

variables, homoscedasticity of error term and accuracy of functional form specification, respectively. The results of these tests are presented in the following tables.

Table S24: Results of evaluation of classical regression assumptions for GDP regression model

Test type	Null hypothesis	Statistics	P-value	Result
jarque–bera test	normal distribution of error terms	0.625	0.731	null hypothesis is not rejected
breusch–godfrey test	there is no serial correlation	2.979	0.06	null hypothesis is not rejected
White test	homoskedasticity	1.935	0.093	null hypothesis is not rejected
Ramsey test	appropriate specification	0.767	0.446	null hypothesis is not rejected

Table S25: Results of evaluation of classical regression assumptions for Total labour force regression model

Test type	Null hypothesis	Statistics	P-value	Result
jarque–bera test	normal distribution of error terms	2.489	0.287	null hypothesis is not rejected
breusch–godfrey test	there is no serial correlation	0.498	0.61	null hypothesis is not rejected
White test	homoskedasticity	2.317	0.069	null hypothesis is not rejected
Ramsey test	appropriate specification	0.427	0.67	null hypothesis is not rejected

Table S26: Results of evaluation of classical regression assumptions for Active population regression model

Test type	Null hypothesis	Statistics	P-value	Result
jarque–bera test	normal distribution of error terms	55.03	0.00	null hypothesis is rejected
breusch–godfrey test	there is no serial correlation	0.549	0.581	null hypothesis is not rejected
White test	homoskedasticity	3.109	0.242	null hypothesis is not rejected
Ramsey test	appropriate specification	0.284	0.777	null hypothesis is not rejected

Table S27: Results of evaluation of classical regression assumptions for Total investment regression model

Test type	Null hypothesis	Statistics	P-value	Result
jarque–bera test	normal distribution of error terms	2.36	0.307	null hypothesis is not rejected
breusch–godfrey test	there is no serial correlation	0.473	0.625	null hypothesis is not rejected
White test	homoskedasticity	0.726	0.581	null hypothesis is not rejected
Ramsey test	appropriate specification	0.691	0.492	null hypothesis is not rejected

Table S28: Results of evaluation of classical regression assumptions for Tax Revenue regression model

Test type	Null hypothesis	Statistics	P-value	Result
jarque–bera test	normal distribution of error terms	1.623	0.444	null hypothesis is not rejected
breusch–godfrey test	there is no serial correlation	2.379	0.103	null hypothesis is not rejected
White test	homoskedasticity	2.02	0.106	null hypothesis is not rejected
Ramsey test	appropriate specification	0.202	0.840	null hypothesis is not rejected

Table S29: Results of evaluation of classical regression assumptions for Government Current Payments regression model

Test type	Null hypothesis	Statistics	P-value	Result
jarque–bera test	normal distribution of error terms	0.609	0.737	null hypothesis is not rejected
breusch–godfrey test	there is no serial correlation	0.49	0.615	null hypothesis is not rejected
White test	homoskedasticity	0.547	0.701	null hypothesis is not rejected
Ramsey test	appropriate specification	0.508	0.613	null hypothesis is not rejected

Table S30: Results of evaluation of classical regression assumptions for Total government expenditure regression model

Test type	Null hypothesis	Statistics	P-value	Result

jarque–bera test	normal distribution of error terms	1.138	0.565	null hypothesis is not rejected
breusch–godfrey test	there is no serial correlation	0.093	0.91	null hypothesis is not rejected
White test	homoskedasticity	1.532	0.182	null hypothesis is not rejected
Ramsey test	appropriate specification	0.585	0.56	null hypothesis is not rejected

Table S31: Results of evaluation of classical regression assumptions for Government Revenue regression model

Test type	Null hypothesis	Statistics	P-value	Result
jarque–bera test	normal distribution of error terms	1.06	0.588	null hypothesis is not rejected
breusch–godfrey test	there is no serial correlation	0.947	0.396	null hypothesis is not rejected
White test	homoskedasticity	0.455	0.86	null hypothesis is not rejected
Ramsey test	appropriate specification	1.592	0.119	null hypothesis is not rejected

Table S32: Results of evaluation of classical regression assumptions for GDP deflator regression model

Test type	Null hypothesis	Statistics	P-value	Result
jarque–bera test	normal distribution of error terms	1.855	0.395	null hypothesis is not rejected
breusch–godfrey test	there is no serial correlation	0.316	0.730	null hypothesis is not rejected
White test	homoskedasticity	0.642	0.718	null hypothesis is not rejected
Ramsey test	appropriate specification	1.854	0.069	null hypothesis is not rejected

Table S33: Results of evaluation of classical regression assumptions for Healthcare Consumer Price Index regression model

Test type	Null hypothesis	Statistics	P-value	Result
jarque–bera test	normal distribution of error terms	4.197	0.122	null hypothesis is not rejected
breusch–godfrey test	there is no serial correlation	0.438	0.04	Null رد hypothesis

White test	homoskedasticity	1.057	0.394	null hypothesis is not rejected
Ramsey test	appropriate specification	1.064	0.292	null hypothesis is not rejected

Table S34: Results of evaluation of classical regression assumptions for Liquidity regression model

Test type	Null hypothesis	Statistics	P-value	Result
jarque–bera test	normal distribution of error terms	0.463	0.793	null hypothesis is not rejected
breusch–godfrey test	there is no serial correlation	1.86	0.166	null hypothesis is not rejected
White test	homoskedasticity	0.831	0.551	null hypothesis is not rejected
Ramsey test	appropriate specification	1.138	0.26	null hypothesis is not rejected

Table S35: Results of evaluation of classical regression assumptions for Out of Pocket Health Expenditure regression model

Test type	Null hypothesis	Statistics	P-value	Result
jarque–bera test	normal distribution of error terms	0.321	0.851	null hypothesis is not rejected
breusch–godfrey test	there is no serial correlation	2.138	0.168	null hypothesis is not rejected
White test	homoskedasticity	0.884	0.582	null hypothesis is not rejected
Ramsey test	appropriate specification	0.776	0.453	null hypothesis is not rejected

Table S36: Results of evaluation of classical regression assumptions for Public Health Insurance Coverage regression model

Test type	Null hypothesis	Statistics	P-value	Result
jarque–bera test	normal distribution of error terms	1.509	0.47	null hypothesis is not rejected
breusch–godfrey test	there is no serial correlation	2.057	0.158	null hypothesis is not rejected
White test	homoskedasticity	1.875	0.137	null hypothesis is not rejected

Ramsey test	appropriate specification	0.844	0.409	null hypothesis is not rejected

Table S37: Results of evaluation of classical regression assumptions for Prepaid Private Health Expenditure regression model

Test type	Null hypothesis	Statistics	P-value	Result
jarque–bera test	normal distribution of error terms	6.518	0.083	null hypothesis is not rejected
breusch–godfrey test	there is no serial correlation	0.533	0.597	null hypothesis is not rejected
White test	homoskedasticity	1.105	0.403	null hypothesis is not rejected
Ramsey test	appropriate specification	0.48	0.637	null hypothesis is not rejected

Table S38: Results of evaluation of classical regression assumptions for Commercial Health Insuran Revenue regression model

Test type	Null hypothesis	Statistics	P-value	Result
jarque–bera test	normal distribution of error terms	1.278	0.527	null hypothesis is not rejected
breusch–godfrey test	there is no serial correlation	1.184	0.337	null hypothesis is not rejected
White test	homoskedasticity	0.829	0.613	null hypothesis is not rejected
Ramsey test	appropriate specification	4.137	0.014	Null hypothesis rejected

Table S39: Results of evaluation of classical regression assumptions for Government Health Expenditure regression model

Test type	Null hypothesis	Statistics	P-value	Result
jarque–bera test	normal distribution of error terms	4.022	0.133	null hypothesis is not rejected
breusch–godfrey test	there is no serial correlation	0.295	0.746	null hypothesis is not rejected
White test	homoskedasticity	0.389	0.932	null hypothesis is not rejected
Ramsey test	appropriate specification	0.231	0.818	null hypothesis is not rejected

Table S40: Results of evaluation of classical regression assumptions for Social Security Organization Health Expenditure regression model

Test type	Null hypothesis	Statistics	P-value	Result
jarque–bera test	normal distribution of error terms	1.239	0.538	null hypothesis is not rejected
breusch–godfrey test	there is no serial correlation	2.263	0.119	null hypothesis is not rejected
White test	homoskedasticity	1.068	0.41	null hypothesis is not rejected
Ramsey test	appropriate specification	0.211	0.834	null hypothesis is not rejected

Table S41: Results of evaluation of classical regression assumptions for Social Security Organization Insurance Coverage regression model

Test type	Null hypothesis	Statistics	P-value	Result
jarque–bera test	normal distribution of error terms	2.527	0.282	null hypothesis is not rejected
breusch–godfrey test	there is no serial correlation	1.079	0.350	null hypothesis is not rejected
White test	homoskedasticity	2.305	0.045	null hypothesis is not rejected
Ramsey test	appropriate specification	1.088	0.283	null hypothesis is not rejected

Table S42 Results of evaluation of classical regression assumptions for Social Security Organization Revenue regression model

Test type	Null hypothesis	Statistics	P-value	Result
jarque–bera test	normal distribution of error terms	4.425	0.109	null hypothesis is not rejected
breusch–godfrey test	there is no serial correlation	0.802	0.455	null hypothesis is not rejected
White test	homoskedasticity	2.207	0.011	null hypothesis is not rejected
Ramsey test	appropriate specification	0.659	0.513	null hypothesis is not rejected

Table S43: The structural macro-econometrics model designed to project Iran’s HCE

$(OHEXJ^{1}_t/HCPI^{2}_t) = \alpha + \beta_1(GDP^{3}_t - \frac{TAXJ^{4}t}{P^{5}t}) + \beta_2 ICOV^{6}_t + \beta_3 PUBHEXJ^{7}t/HCPI_t + \beta_4 POP60R^{8}_t + HCPI_0{}^{9}t + \beta_6(OHEXJ_{t-1}/HCPI_{t-1})$
$ICOV_t = \alpha + \beta_1 GDP_t + \beta_3 POPT^{10} + ICOV_{t-1}$
$(PPHEXJ^{11}_t/HCPI_t) = \alpha + \beta_1 GDP_t + \beta_2 POPT_t + \beta_3(PIRJ^{12}_t/P_t) + \beta_4(PPHEXJ_{t-1}/HCPI_{t-1})$
$(PIRJ_t/P_t) = \alpha + \beta_1 GDP_t + \beta_2(PIRJ_{t-1}/P_{t-1})$
$(PHEXJ^{13}_t/HCPI_t) = (OHEXJ_t/HCPI_t) + (PPHEXJ_t/HCPI_t)$
$(SHIJ^{14}_t/HCPI_t) = \alpha + B_1 POP60R_t + \beta_2 SICOV^{15}_t + \beta 3(SIRJ^{16}_t/P_t) + \beta 4(SHIJ_{t-1}/HCPIt-1)$
$(SIRJ_t/P_t) = \alpha + \beta_1 GDP_t + \beta_2 L^{17}_t + \beta_3(XORJ^{18}_t/P_t) + \beta_5(SIRJ_{t-1}/P_{t-1})$
$SICOV_t = \alpha + \beta_1 GDP_t + \beta_2 L_t + \beta_3 POPT + SICOV_{t-1}$
$(GHEXJ^{19}_t/HCPI_t) = \alpha + B_1 POP60R^{20}_t + \beta_2 POP15R_t + \beta_3(GRJ^{21}_t/P_t) + \beta_4 URR^{22}_t + \beta_5(GHEXJ_{t-1}/HCPI_{t-1})$
$(PUBHEXJ_t/HCPI_t) = (GHEXJ_t/HCPI_t) + (SHIJ_t/HCPI_t)$
$(THEXJ^{23}_t/HCPI_t) = (PUBHEXJ_t/HCPI_t) + (PHEXJ_t/HCPI_t + (FHEXJ^{24}_t/HCPI_t)$
$GDP_t = \alpha + \beta_1 L_t + \beta_2 K^{25}_t + \beta_3 GDP_{t-1}$
$L_t = = \alpha + \beta_1(W^{26}_t/CPI^{27}_t) + \beta_2 F^{28}_t + \beta_3 L_{t-1}$
$F_t = = \alpha + \beta_1 POP2060^{29}_t + \beta_2 F_{t-1}$
$CPI_t = = \alpha + \beta_1 P_t + \beta_2 CPI_{t-1}$
$W_t = = \alpha + \beta_1 W_{t-1}$
$k = 0.955 * k(-1) - 0.378527 * wd^{30} - 0.153215 * erd^{31} + i^{32}$
$I_t = = \alpha + \beta_1(GDP_t) + \beta_2 R^{33}_t + \beta_3 I_{t-1}$
$(TAXJ_t/P_t) = \alpha + \beta_1 GDP + \beta_2(TAXJ_{t-1}/P_{t-1})$
$(GRJ_t/P_t) = \alpha + B_1(TAXJ_t/P_t) + B_2(XORJ_t/P_t) + B_3(GRJ_{t-1}/P_{t-1})$
$(GEJ^{34}_t/P_t) = \alpha + B_1(TAXJ_t/P_t) + B_2(XORJ_t/P_t) + B_3(GEJ_{t-1}/P_{t-1})$
$P_t = \alpha + \beta_1 M_2 J^{35}_t + \beta_2 GDP_t + \beta_3 P_{t-1}$
$HCPI_t = \alpha + \beta_1 M_2 J_t + \beta_2 GDP_t + \beta_3 HCPI_{t-1}$
$M_2 J_t = \alpha + \beta_1 XOG^{36}_t + \beta_2 GEJ_t + \beta_3 M_2 J_{t-1}$

[1] Out of Pocket Health Expenditure in current price
[2] Healthcare Consumer Price Index
[3] Gross Domestic Product in real price
[4] Government Tax Revenue in current price
[5] GDP deflator
[6] Public Health Insurance Coverage
[7] Public Health Expenditure in current price
[19] Government Health Expenditure in current price
[20] Share of population < 15 years
[21] Government Revenue in current price
[22] Urbanization rate
[23] Total Health Expenditure in current price
[24] Foreign Health Expenditure in current price
[25] Capital accumulation of all sectors of the economy

[8] Share of population > 60 years
[9] Inflation rate
[10] Total Population
[11] Prepaid Private Health Expenditure in current price
[12] Commercial Health Insuran Revenue
[13] Private Health Expenditure in current price
[14] Social Security Organization Health Expenditure in current price
[15] Social Security Organization Insurance Coverage
[16] Social Security Organization Revenue
[17] Total labour force
[18] Oil and Gas revenue
[26] Real wage index
[27] Consumer Price Index
[28] Active population
[29] 20 years <Share of population< 60 years
[30] Destruction of capital caused by the imposed war
[31] Destruction of capital caused by Rudbar earthquake
[32] Total investment
[33] Interest rates on long-term deposits
[34] Government Current Payments in current price
[35] Liquidity in current price
[36] Oil and Gas Exports(mollion $)

2. Evaluating the validity of the final dynamic macro-structural dynamic econometric model

RMSE and Tile indices along with dynamic simulation are used in order to assess final sytem validity. Dynamic simulation on the one hand provides a criterion for assessing the sytems validity and on the other hand, could be used to assess the consequences and results of different policies implementations. Dynamic simulation is a very difficult test for assessing the validity of macrostructural econometric sytems. In this type of simulation, the data related to the pattern endogenous variables are initially inserted in the sytem and the sytem generates the sytem variables (simulated trend of variables), without knowing the real variables values. Subsequently, the values generated by the dynamic model could be compared with the real values, through drawing a diagram. The similarity rate of these two diagrams is one of the assessment criteria in validity test of the dynamic system. Very few dynamic systems could pass this difficult test successfully. Graphic drawings of this comparison are provided for some of the main variables in the results of the article(Figure 1).

In addition to graphical drawing, quantitative criteria such as U Tile inequality index and RMSE index have been used to evaluate the validity of the model in simulating each of the dependent variables(Table).

Table S44: RMSE and U Tile indeces of dependent variables in developed structural macro-econometrics model

Dependent variables	U tile index	RMSE
GDP	0.05	5.57
Total labour force	0.02	2.32
Active population	0.02	2.32
Consumer Price Index	0.20	31.44
Total investment	0.09	15.27
Capital accumulation of all sectors of the economy	0.03	4.07
Government Tax Revenue	0.15	26.48
Government Current Payments	0.167	2.23
Total government expenditure	0.22	29.45
Government Revenue	0.18	21.95
GDP deflator	0.24	24.35
Healthcare Consumer Price Index	0.11	27.96
Liquidity	0.09	9.50
Out of Pocket Health Expenditure	0.10	1.83
Public Health Insurance Coverage	0.05	5.55
Prepaid Private Health Expenditure	0.13	37.53

Commercial Health Insuran Revenue	0.19	30.96
Private Health Expenditure	0.10	12.06
Government Health Expenditure	0.08	14.53
Social Security Organization Health Expenditure	0.06	7.72
Social Security Organization Insurance Coverage	0.06	5.93
Social Security Organization Revenue	0.09	9.09
Public Health Expenditure	0.07	11.51
Total Health Expenditure	0.07	9.57
Total Health Expenditure as % of GDP	0.04	4.98
Public Health Expenditure as % of Total Health Expenditure	0.05	5.69
Private Health Expenditure as % of Total Health Expenditure	0.07	7.55
Out of Pocket Health Expenditure as % of Total Health Expenditure	0.06	5.98
Government Health Expenditure as % of Total Health Expenditure	0.09	9.29
